# Characteristics of antibiotic resistance mechanisms and genes of *Klebsiella pneumoniae*

**DOI:** 10.1515/med-2023-0707

**Published:** 2023-05-12

**Authors:** Yanping Li, Suresh Kumar, Lihu Zhang, Hongjie Wu, Hongyan Wu

**Affiliations:** Pharmacy Department, Jiangsu Vocational College of Medicine, 224005 Yancheng, Jiangsu Province, China; Post Graduate Centre, Management and Science University, University Drive, Off Persiaran Olahraga, Section 13, 40100, Selangor, Malaysia; Department of Diagnostic and Allied Health Science, Faculty of Health and Life Sciences, Management and Science University, Shah Alam, Malaysia; School of Electronic and Information Engineering, Suzhou University of Science and Technology, Suzhou, China

**Keywords:** *Klebsiella pneumoniae*, antibiotic resistance, resistance mechanisms

## Abstract

*Klebsiella pneumoniae* is an important multidrug-resistant (MDR) pathogen that can cause a range of infections in hospitalized patients. With the growing use of antibiotics, MDR *K. pneumoniae* is more prevalent, posing additional difficulties and obstacles in clinical therapy. To provide a valuable reference to deeply understand *K. pneumoniae*, and also to provide the theoretical basis for clinical prevention of such bacteria infections, the antibiotic resistance and mechanism of *K. pneumoniae* are discussed in this article. We conducted a literature review on antibiotic resistance of *K. pneumoniae.* We ran a thorough literature search of PubMed, Web of Science, and Scopus, among other databases. We also thoroughly searched the literature listed in the papers. We searched all antibiotic resistance mechanisms and genes of seven important antibiotics used to treat *K. pneumoniae* infections. Antibiotics such as β-lactams, aminoglycosides, and quinolones are used in the treatment of *K. pneumoniae* infection. With both chromosomal and plasmid-encoded ARGs, this pathogen has diverse resistance genes. Carbapenem resistance genes, enlarged-spectrum β-lactamase genes, and AmpC genes are the most often β-lactamase resistance genes. *K. pneumoniae* is a major contributor to antibiotic resistance worldwide. Understanding *K. pneumoniae* antibiotic resistance mechanisms and molecular characteristics will be important for the design of targeted prevention and novel control strategies against this pathogen.

## 1 Introduction

The gram-negative bacterium *Klebsiella pneumoniae* is a member of the family *Enterobacteriaceae*, closely related to the well-known *Salmonella enterica* and *Escherichia coli* pathogens [1]. *K. pneumoniae* can ferment lactose and has capsular polysaccharides. *K. pneumoniae* is a common hospital-acquired opportunistic pathogen, accounting for about 30% of all gram-negative bacterial infections.

**Figure 1:**
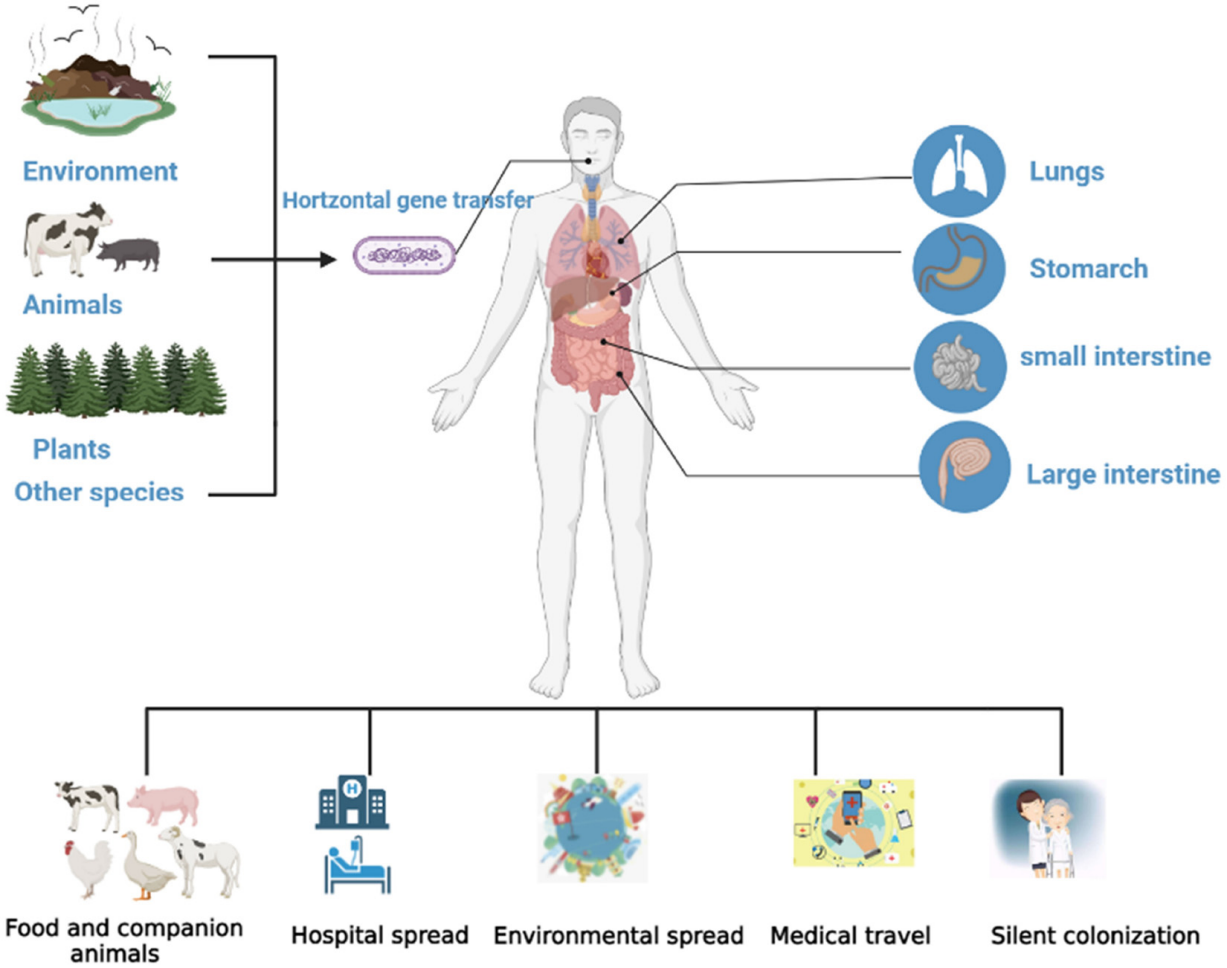
Transmission of *K. pneumoniae.*

*K. pneumoniae* can be commensals in a range of environments, including soil, water, a variety of plants, insect species, birds, and animals. Typical *K. pneumoniae* is widely distributed among human and animal mouth, skin, respiratory tract, urogenital tract, and intestine [2,3]. *K. pneumoniae* causes infections through gene or plasmid horizontal transfer [4]. A large percentage of *K. pneumoniae* infections occur in newborns, the elderly, and those with compromised immune systems [2]. It can infect the respiratory tract, the urinary tract, as well as wounds or soft tissues. Even with appropriate antibiotic treatment, the mortality rate of hospital-acquired pneumonia is still more than 50%. The incidence rate and mortality of diseases caused by *K. pneumoniae* are very high, especially for newborns, leukaemia patients, and other immunodeficiency patients. With the growing use of antibiotics, multidrug-resistant (MDR) *K. pneumoniae* has become more common, posing greater difficulties and obstacles in clinical treatment. The World Health Organization recognizes extended-spectrum β-lactam (ESBL)-producing and carbapenem-resistant *K. pneumoniae* (CRKP) as a critical public health threat [5]. Transmission of *K. pneumoniae* is shown in Figure 1.

We deepened all antibiotic resistance mechanisms and genes of seven important antibiotics used to treat *K. pneumoniae* infections. We conducted a literature evaluation of antibiotic resistance in *K. pneumoniae.* We ran a thorough literature search in PubMed, Web of Science, and Scopus, among other databases. Keywords such as antibiotics or antibiotic resistant or antimicrobial resistant or drug resistant or drug-resistant were searched in various databases, and literatures with keywords such as *K. pneumoniae* or *K. pneumoniaee* were also included. It also contains seven antibiotics, namely *β*-lactam, macrolide, aminoglycoside, lincomycin, chloramphenicol, peptides, and other themes. We also thoroughly deepened the literature listed in the papers.

## 2 Antibiotic resistance in *K. pneumoniae*

Antibiotics such as aminoglycosides and cephalosporins are commonly used to treat *K. pneumoniae*. The choice of an antimicrobial agent is based on the patient’s health, medical history, and disease severity [9,12]. For urinary tract infections caused by MDR-resistant *Klebsiella* species, a combination of amikacin and meropenem has been suggested [6]. *Klebsiella* infections have caused liver fistulas in patients with diabetes mellitus in Taiwan, and third-generation carbapenems have been used to treat them. For patients in clinical settings, antimicrobial resistance (AMR) in MDR *K. pneumoniae* is a major public health concern, restricting treatment options [7]. When compared to individuals who received combination therapy, those who received monotherapy had more treatment failures (49% vs 25%; *p* = 0.01) [8].

Combination therapy can delay the emergence of resistance because the simultaneous use of multiple mechanisms of action increases the pharmacodynamic killing activity of antibiotics [9]. Combination therapy with carbapenems, tetracyclines, polymyxins, and fosfomycin is suggested and frequently utilized due to the increased degree of AMR in *K. pneumoniae* and the rising incidence of CRKP. Repeated exposure to a large range of antimicrobial compounds can trigger the emergence of new MDR phenotypes. With the wide abuse of β-lactam antibiotics and carbapenems in clinical practice, the detection rate of *K. pneumoniae* infection as an opportunistic pathogen is gradually increasing in clinical practice.

**Figure 2:**
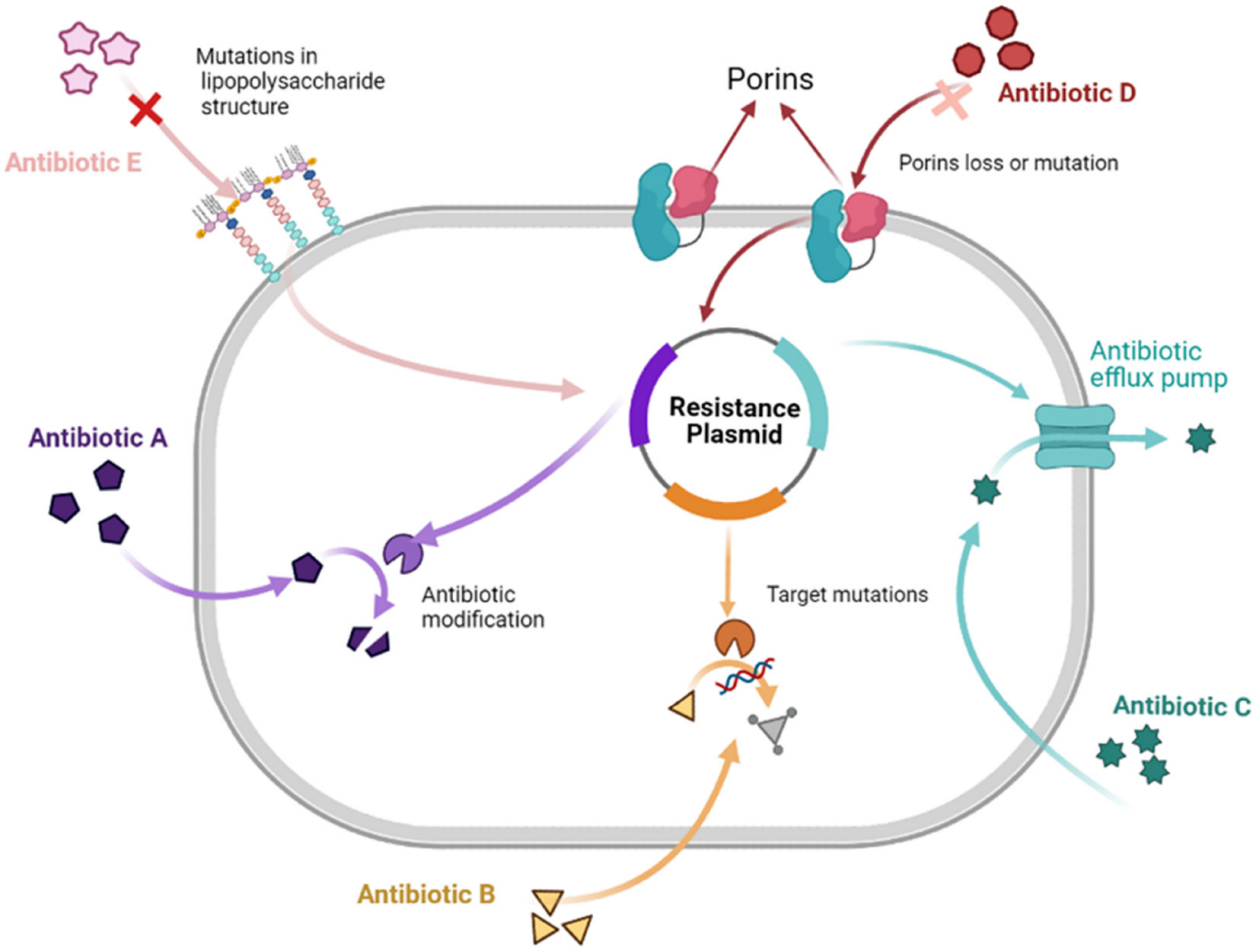
Various mechanisms conferring antibiotic resistance to *K. pneumoniae.*

*K. pneumoniae* shows resistance against the main antibiotic classes: carbapenems, cephalosporins, aminoglycosides, and fosfomycin, leading to the therapeutic failure of these agents [10]. The development of antibiotic resistance in *K. pneumoniae* has led to a decline in the effectiveness of traditional treatments against the pathogen. Resistance may occur due to increased efflux, drug inactivation, or altered binding to the target site. Many strains of *K. pneumoniae* produce ESBL or form biofilms, further exacerbating resistance. The antibiotic resistance of *K. pneumoniae* is mainly produced in the following five ways: (1) enzymatic antibiotic inactivation and modification, (2) antibiotic target alteration, (3) porin loss and mutation, (4) increased efflux pump expression of the antibiotic, and (5) biofilm formation [11,12]. The five mechanisms conferring antibiotic resistance to *K. pneumoniae* are shown in Figure 2 and Table 1.

**Table 1:** Resistant strategies in *K. pneumoniae*

Resistant strategies in *K. pneumoniae*	Key findings	References
Enzymatic antibiotic inactivation and modification	β-Lactamase is an important resistance mechanism, which is divided into ESBLs, AmpC, and carbapenemases	[13,18]
Antibiotic targets alteration	*K. pneumoniae* causes drug resistance by mutating the target gene or methylating some bases	[19,20,21]
Porin loss and mutation	*K. pneumoniae* develops resistance by reducing the entry of antimicrobial agents into the bacteria by reducing the outer membrane pore protein	[25,26]
Increased efflux pump expression of the antibiotic	Efflux pumps reduce intracellular drug concentrations by releasing antimicrobial cells outside the cell, thereby reducing susceptibility to multiple antibiotics	[29]
Biofilm formation	Biofilms have osmotic barrier properties and are resistant to antimicrobial agents	[31,32,33]

## 3 Enzymatic antibiotic inactivation and modification

Drug alteration is a major mechanism of resistance against antibiotics *in K. pneumoniae* [13]. β-Lactamase is an important resistance mechanism, which hydrolyses the β-loop of β-lactam. β-Lactamases are divided into ultra-broad-spectrum β-lactamases (ESBLs), cephalosporinases (AmpC), and carbapenemases [14,15,16,17]. The expression of these enzymes in *K. pneumoniae* renders it resistant to penicillins, cephalosporins, and carbapenems. ESBLs include SHV, TEM, OXA, CTX, and other types. AmpC is resistant to cephalosporins, cephalomycin, and enzyme inhibitors of the first to the third generation, which can be mediated by chromosomes or plasmids. Up to now, there are more than 40 genotypes of AmpC enzyme, which can spread rapidly among strains by the plasmid. The production of carbapenemases decreases the sensitivity of *K. pneumoniae* to carbapenems, and the emergence of CRKP makes the treatment difficult. According to the Ambler classification, carbapenemases can be classified into classes A, B, and D [18].

## 4 Antibiotic targets alteration

Fluoroquinolone antibiotics target DNA topoisomerase [19]. Aminoglycoside antibiotics target 16S rRNA. The mechanism of resistance to polymyxin in *K. pneumoniae* usually involves the modification of lipid A [20], and the mechanism of resistance to fosfomycin involves the modification targeting MurA [21]. *K. pneumoniae* causes drug resistance by mutating the target gene or methylating some bases so that the corresponding antimicrobial agents cannot bind to the target site.

## 5 Porin loss and mutation

*K. pneumoniae* develops resistance by reducing the entry of antimicrobial agents into the bacteria by reducing the outer membrane pore protein. Outer membrane proteins (OMPs) or porins are trimeric transmembrane proteins that are abundantly expressed on the outer membranes of Gram-negative bacteria [22,23,24]. In *K. pneumoniae*, OmpK35 and OmpK36 are the two major nonspecific porins associated with AMR. LamB, OmpK26, PhoE, and KpnO porins also contribute to intrinsic resistance [25,26].

## 6 Increased efflux pump expression of the antibiotic

Efflux pumps are membrane proteins involved in substance expulsion that reduce intracellular drug concentrations by releasing antimicrobial cells outside the cell, thereby reducing susceptibility to multiple antibiotics [27,28]. The active efflux system AcrAB-TolC can exocytosis many kinds of antibiotics, including β-lactam, macrolides, fluoroquinolones, and tetracycline, which is an important reason for the MDR *K. pneumoniae* [29,30].

## 7 Biofilm formation

*K. pneumoniae* is prone to form biofilms, and structures such as capsular and pili play an important role in the formation of biofilms [31]. Biofilms have osmotic barrier properties and are resistant to antimicrobial agents, and one study showed that *K. pneumoniae* biofilms reduced sensitivity to gentamicin, ampicillin, and ciprofloxacin [32]. Colistin resistance has also been linked to biofilm formation [33].

## 8 Resistance mechanisms and genes

β-Lactam antibiotics are frequently used to treat *K. pneumoniae* infections. When patients are infected with *K. pneumoniae* that is MDR or extended drug-resistant, they have no choice but to use other antibiotics (aminoglycosides, quinolones, polymyxins, tigecycline, etc.). However, when these antibiotics are used in clinical settings, they can lead to drug resistance. Antibiotic resistance-related genes were carefully summarized, and their functions in *K. pneumoniae* are systematically presented in Table 2.

**Table 2:** Antibiotic resistance-related genes in *K. pneumoniae*

Characteristic	Gene name	Gene functions	References
β-Lactam	*bla*SHV, *bla*TEM, *bla*CTX	ESBLs	[34,35,36]
	*bla*GES, *bla*SFO, *bla*PER, *bla*TLA, *bla*VEB, *bla*KLUC-5	Lateral gene transfer	[37,38,39,40]
	*bla* KPC *Bla* NDM, *bla* VIM, *bla* IMP, and *bla* OXA	Carbapenemase	[41,42]
	*bla*CMY, *bla*DHA, *bla*FOX, *bla*MOX	AmpC plasmids	[43,44]
Aminoglycoside	aac, ant, aph gene,16S rRNA methylase	Plasmid-encoded	[45,46,47,48]
	AcrAB-TolC, kpnEF, KpnO	Efflux pump systems	[49]
Quinolone	DNA gyrase, topoisomerase IV	Quinolone-binding targets	[50]
	OmpK36, acrAB, kdeA, OqxAB, aa(6’)-Ib-cr	PMQR	[51,52,53,54]
Polymyxin	phoPQ, pmrA, pmrD, and mgrB	Regulative gene	[55,56,57]
	mcr-1	Via plasmid	[58,59,60]
Tigecycline	AcrAB-TolC, OqxAB	Efflux pump systems	[61]
	RarA, RamA, RamR, and AcrR	Regulators of efflux pumps	[61]
	rpsJ	Encoding ribosome	[62]
	tetA	Efflux pump systems	[61]
Fosfomycin	fos	Via plasmid	[63]

## 9 β-Lactamase Resistance Genes

β-Lactamase produced by *K. pneumoniae* hydrolyses the β-lactam ring in antibiotics, resulting in resistance to β-lactam antibiotics. *K. pneumoniae* is naturally resistant to numerous β-lactamase genes attributed to the prevalence of the SHV β-lactamase in the genome sequence, and ampicillin resistance is a defining trait of the organism.

## 10 ESBLs

ESBLs are plasmid-based antibiotic resistance pathways identified in the accessory genome. In Germany [35], the blaSHV-2 (ESBL) gene in *K. pneumoniae* was found for the first time. Soon after, blaTEM-3, a viral vector ESBL mutant gene, was found in France [64]. The enlarged-spectrum action of ESBL genes against β-lactams, including third-generation carbapenems, is inhibited by clavulanic acid [65].

*K. pneumoniae* that produces ESBL has become a prevalent pathogen in hospital infection outbreaks. CTX-M gradually superseded TEM and SHV as the main genotype of ESBLs owing to the accessibility of plasmids and transposons generating *bla*CTX-M-type ESBLs [36]. Other ESBL genotypes were also transmitted to *K. pneumoniae* by horizontal gene transfer, including *bla*OXA type ESBLs [38] and the uncommon genes *bla* GES, *bla* SFO [37], or *bla* PER, *bla* TLA, *bla* VEB [40], and *bla* KLUC-5 [39]. *K. pneumoniae* that produces ESBL is becoming more common over the world, with endemic rates of up to 50% in some areas [39]. Carbapenems have traditionally been the treatment of choice for treating ESBL-producing bacterial infections.

## 11 Carbapenem resistance genes

Carbapenem use has increased significantly as a result of the MDR phenotypic characteristics of ESBL-producing *K. pneumoniae* strains. Carbapenem resistance has evolved, possibly as a result of the selective pressure of carbapenems treatment, and *K. pneumoniae* has emerged as the most prevalent carbapenem-resistant *Enterobacteriaceae* (CRE).

The carbapenem enzymes regulated by plasmids are still the most concerned pathway of multidrug resistance. KPC is a serine-based class β-lactamase that is the most common and damaging carbapenemase in *K. pneumoniae*. Clonal group 258 (CG258) is linked to KPCs [66,67]. ST258 (ST258) is found in Europe, America, and Asia, while ST11 is prevalent in Asia [68,69,70,71,72]. *bla* KPC genes are discovered in a specific Tn4401 transposition form and are incorporated onto plasmids of several plasmid types in addition to clonal dissemination [73], making it easier to spread the gene to others [74]. *Bla* NDM, *bla*_VIM_, *bla*_IMP_, and *bla* OXA are other carbapenemase genes found in *K. pneumoniae* [41]. Ripabelli et al. evaluated resistance to 19 antibiotics in Italy by disk diffusion and agar dilution method. The highly pathogenic variant of NDM-1 was screened for the first time in their study [75]. Such resistance genes can cause a large number of carbapenemase-producing *Enterobacteriaceae* (CPE) to be resistant to many commonly used clinical antibiotics, resulting in the difficult clinical treatment of CPE and high mortality. KPCs are generally resistant to conventional β-lactamase inhibitors, creating a therapeutic issue [76]. These resistances are virtually impossible to regulate due to the translocation of carbapenemase-encoding genes from *K. pneumoniae* plasmids onto the chromosome [41]. In the lack of the carbapenemase gene, *K. pneumoniae* can become carbapenem-resistant, owing to the loss of porin, increased effluent pump, and excessive production of *β*-lactamases such as ESBL and AmpC. Clinically, CRKP infection is a tough problem in the clinic [77].

## 12 Plasmid-mediated AmpC Genes

Plasmid-mediated AmpC-like cephalosporins evolved and dispersed in these species due to *K. pneumoniae’*s exceptional versatility in adding *β*-lactamase genes onto transportable plasmids that facilitate the dissemination [43,44]. The *bla* AmpC gene sequences *bla* CMY, DHA, FOX, and MOX are most frequent in *K. pneumoniae*. *K. pneumoniae* had better *β*-lactam resistance owing to the presence of *bla* AmpC coupled with gene encoding losses or enhanced efflux, similar to *bla*ACT-1. Plasmid genes can be readily abundantly expressed on plasmids due to the increase of many copies or promoter strength, resulting in carbapenem resistance [44].

Multiple-lactamase genes, including AmpC, KPC, SHV, and *β*-lactamase inhibitors, may be present in some *K. pneumoniae* strains. Multiple-resistant genes carried by the same strain have synergistic effects. For instance, while NDM, Vim, and IMP are not resistant to monocyclic antibiotics like aztreonam, they may develop resistance to aztreonam if ESBL or AmpC is present.

## 13 Aminoglycoside resistance genes

Aminoglycosides were commonly used in antibacterial chemotherapy from 1940 to 1980 until third-generation cephalosporins, carbapenems, and fluoroquinolones replaced them [78]. *K. pneumoniae* acquired the primary antibiotic resistance mechanisms during this time, including drug-modifying enzymes with varied functions, such as adenylation, acetylation, or phosphorylation as well as all transposon resistance genes from the *aac, aph,* and *ant* gene families [45].

The use of aminoglycosides was limited, which slowed down the emergence of novel resistance genes till the armA gene family expressed 16S rRNA methylase [46]. In *K. pneumoniae*, these genes are plasmid-encoded, and while drug-modifying enzymes inhibit activity [48], 16S rRNA methylase is resistant to almost all aminoglycosides, including plazomicin and newly discovered aminoglycosides [47].

Genes on chromosomes also have a role in the *K. pneumoniae* resistance to aminoglycoside antibiotics, which modify cell permeability through changes in the *AcrAB-TolC* and *KpnEF* efflux pump systems, as well as the loss of the putative porin *KpnO*. The *AcrAB-TolC* and *KpnEF* efflux pump systems changed throughout time, resulting in variable levels of resistance to different aminoglycoside antibiotics. Tobramycin and gentamicin resistance was predominant in the former, whereas tobramycin and vancomycin resistance was predominant in the latter, with gentamicin and streptomycin resistance being minor. This implies that various aminoglycosides correspond to various cell channels. Resistance to tobramycin, streptomycin, and spectinomycin was linked to the loss of the pore protein *KpnO* [49].

## 14 Quinolone resistance genes

Quinolone antibiotics function by inhibiting topoisomerases, which hinder DNA replication in bacteria. Mutations in the target gene increased MDR efflux production, and mutations to enzymes and proteins all contribute to *K. pneumoniae’s* tolerance to fluoroquinolones [79]. Topoisomerase IV and DNA gyrase are quinolone-binding targets with chromosomal resistance mechanisms. ParC and gyrA *K. pneumoniae* mutations were found earlier and also more frequently [50]. Changes in cell permeability in *K. pneumoniae* were linked to drug-resistant strains.

Among the most common are the deficiency of OmpK36 [51], overexpression of the gene acrAB [52], and nonalteration production of kdeA [53]. OqxAB is found in many bacteria and has been linked to plasmid-mediated quinolone resistance (PMQR) [80]. *K. pneumoniae* quinolone resistance has also been linked to efflux pump regulators [81].

The PMQR determinant, which is found in *K. pneumoniae* and other *Enterobacteriaceae* species, is another type of quinolone resistance gene. These genes encode a protein family that protects DNA gyrase and topoisomerase IV from quinolones. In *K. pneumoniae* [82], aa(6’)-Ib-cr, another PMQR gene, is thought to be the only one involved in quinolone modification. It can inactivate limited quinolones that contain the enzyme’s substrate, as well as other antibiotics. It was recently discovered on chromosomes as well. PMQR gene expression provides mechanisms for low or moderate quinolone resistance, but it also creates favourable conditions for chromosomal genetic changes to emerge [83].

## 15 Polymyxin resistance gene

The recent appearance of CRE has necessitated a reintroduction of *polymyxins* as a last-line treatment [84]. Polymyxin resistance in *K. pneumoniae* is typically induced by alterations in regulative genes, for instance, *mgr*B, which regularizes the changes of bacterial lipid A, a target of polymyxin antibiotics, lowering polymyxin interaction [55,56,57].

In 2016, the *mcr*-1 gene conferred colistin resistance via plasmid in an *E. coli* strain from China [85]. This study illustrates that easily transmissible genes potentially result in pan-resistance. In China, mcr-1 is rarely discovered in *K. pneumoniae* BSI isolates and is more commonly seen in *E. coli.* The first mcr-1 case was discovered in America in 2016. A pan-resistant isolate of *K. pneumoniae* was discovered in September 2016, although colistin resistance was not mediated by *mcr-1* in this isolate [58,59,60].

## 16 Tigecycline resistance genes

Tigecycline, as a new tetracycline antibiotic, has a broad-spectrum activity against ESBL-producing strains [86]. It has been accustomed to healing *K. pneumoniae* infection since 2005 and the tigecycline resistance in *K. pneumoniae* was reported shortly after its first use. It is known that the resistance gene of this antibiotic is located on the chromosome, and the mechanism includes the modification of 30S and 16S ribosomal targets of antibiotics and the alteration of cell permeability [61]. The mechanism of antibiotic resistance is mainly related to the Ade-ABC efflux pump, Oqx-AB efflux pump, KpgABC efflux pump, Tet (A) mutant, and ribosomal protein.

Active efflux pump widely exists in the genome of *K. pneumoniae*. It can selectively or nonselectively pump the drugs or substrates in the bacteria out of the body, resulting in the decrease of antibacterial drug concentration in the body and drug resistance. The efflux pump transport systems involved in the resistance of *K. pneumoniae* to tigecycline are the AcrAB-TolC efflux pump, OqxAB efflux pump, KpgABC efflux pump, and Tet (A) efflux pump variants. Among them, the AcrAB TolC efflux pump, OqxAB efflux pump, and KpgABC efflux pump belong to the resistance nodule cell division family, and Tet (A) efflux pump variant belongs to the major facilitator super superfamily.

Ribosomal protein S10 is encoded by the *rpsJ* gene and is a component of the ribosomal 30S subunit. It is located near the main binding site of tetracycline and tigecycline in the ribosomal 30S subunit. Villa et al. [62] obtained three *K. pneumoniae*-resistant strains of tigecycline. One strain indicated that the coding gene *rpsJ* of S10 ribosomal protein adjacent to the target of tigecycline in the ribosomal 30S subunit had a point mutation. The *reps* mutation alone could confer tigecycline resistance to *Enterococcus faecalis* and conducted an adaptability test on six common clinical pathogens [87]. Therefore, the structural change of ribosomal protein S10 is also a potential new mechanism, which deserves attention in the follow-up research. Lupien et al. [88] show that in addition to S10, ribosomal proteins S3 and S13 are also located near the binding domain between tetracycline and ribosomal subunit, and S3 has been proved to have the function of maintaining the structural integrity of the tetracycline-binding site. Similarly, it is inferred that the structural mutation of the S3 protein may also result in tigecycline resistance. Studies have shown that without the involvement of efflux pump, *rpsJ* gene mutation can lead to specific resistance to tigecycline.

## 17 Fosfomycin resistance genes

Fosfomycin was discovered in 1969 and has a wide range of bactericidal activities [89]. Although fosfomycin is an old antibiotic, it has received renewed interest and is increasingly being used to treat infections caused by MDR bacteria [90]. However, with the increasing use of fosfomycin, resistant strains are being reported [91,92]. Resistance mechanisms of fosfomycin have been reported, including amino acid replacement or overexpression of the fosfomycin target protein MurA, deficient or reduced expression of two transporters (GlpT and UhpT), and the presence of the fos gene encoding a fosfomycin-modified enzyme that inactivates fosfomycin by activating glutathione S-transferase activity [93]. Liu et al. reported that the fosA3 gene is the main mechanism of the resistance of CRKP to fosfomycin, which can be transmitted by plasmid in hospitals. Fosfomycin target protein MurA and glpT transporter mutations were found in fosA3-negative CRKP with fosfomycin resistance [63].

## 18 Other mechanisms

Tolerance and persistence have long been recognized as helping bacteria survive antibiotic exposure [94]. Persister cells (persistence phenotype) with an epigenetic feature that allows them to be resistant to antibiotics while remaining latent and metabolically inactive [95].

Changes in the number of certain proteins, metabolites, and signal transduction, such as toxic chemical modules, adenosine triphosphate, and guanosine (penta) tetraphosphate, have been associated with the creation of persister formation. Despite contradicting changes in proteins, metabolites, and signal transduction, persistent bacteria form as a result of sluggish growth alone, according to Pontes and Groisman [96]. Persister cells have been seen in bacterial populations before antibiotics were introduced, sluggish growing or quiescent due to phenotypic switching [97,98]. After the antibiotics are removed, the surviving persisters regenerate into a new heterogeneous population with tolerant and sensitive subpopulations, much like the initial culture [99]. Increased antibiotic concentrations and longer antibiotic treatment reduced *K. pneumoniae* persistence [100]. In the fight against MDR, understanding the molecular processes governing bacterial tolerance and persistence phenotypes is critical, as it will enable the identification of new targets for creating novel anti-infective treatments.

## 19 Conclusions

In this study, the antibiotic resistance status, antibiotic resistance mechanism, and resistance genes of *K. pneumoniae* were described. In the resistance mechanism, ESBLs, carbapenemase, or AmpC targets alteration, porin loss and mutation, efflux pump overexpression, and horizontal dissemination of mobile gene elements were also studied in many fields. Up to now, the mechanism of antibiotic resistance of *K. pneumoniae* has not been thoroughly studied in many aspects, such as how biofilm formation regulates antibiotic resistance. Addressing the escalating prevalence of AMR, antibacterial drug therapy effect weakened, clinical treatment of severe problems such as no cure, the new drug-resistant bacteria drugs research and development work is imminent.

Novel therapies like phage therapy, nanoparticles, phytotherapy, photodynamic therapy, and antimicrobial peptides are being used to overcome resistance in *K. pneumoniae* infections [101,102,103,104,105]. The mechanisms of antibiotic resistance of *K. pneumoniae* are complex and diverse. We should provide insights into useful strategies to combat this important pathogen. How to prevent and to treat infection has become an urgent problem to be solved. It is important to determine the main antibiotic resistance genotypes for the rational use of antibiotics. Understanding *K. pneumoniae* antibiotic resistance mechanisms and molecular characteristics will be important for the design of targeted prevention and novel control strategies against this pathogen. At the same time, to effectively reduce and control the generation and spread of MDR bacteria, we should actively carry out antibiotic resistance monitoring and timely grasp the mechanism and characteristics of antibiotic resistance.

## References

[1] Adeolu M, Alnajar S, Naushad S, S. Gupta R. Genome-based phylogeny and taxonomy of the ‘Enterobacteriales’: proposal for Enterobacterales ord. nov. divided into the families Enterobacteriaceae, Erwiniaceae fam. nov., Pectobacteriaceae fam. nov., Yersiniaceae fam. nov., Hafniaceae fam. nov., Morganellaceae fam. nov., and Budviciaceae fam. nov. Int J Syst Evol Microbiol. 2016;66(12):5575–99. 10.1099/ijsem.0.001485 27620848

[2] Podschun R, Ullmann U. Klebsiella spp. as Nosocomial Pathogens: Epidemiology, Taxonomy, Typing Methods, and Pathogenicity Factors. Clin Microbiol Rev. 1998;11(4):589–603. 10.1128/cmr.11.4.589 PMC88898 9767057

[3] Bagley ST. Habitat association of Klebsiella species. Infect Control. 1985;6(2):52–8. 10.1017/s0195941700062603 3882590

[4] Wyres KL, Holt KE. Klebsiella pneumoniae as a key trafficker of drug resistance genes from environmental to clinically important bacteria. Curr OpMicrobiology. 2018;45:131–9. 10.1016/j.mib.2018.04.004 29723841

[5] Shrivastava S, Shrivastava PS, Ramasamy J. World health organization releases global priority list of antibiotic-resistant bacteria to guide research, discovery, and development of new antibiotics. J Med Soc. 2018;32(1):76.

[6] Yasin F, Assad S, Talpur AS, Zahid M, Malik SA. Combination therapy for multidrug-resistant klebsiella pneumoniae urinary tract infection. Cureus. 2017;9(7):e1503. 10.7759/cureus.1503 PMC5608481 28948123

[7] Bassetti M, Righi E, Carnelutti A, Graziano E, Russo A. Multidrug-resistant Klebsiella pneumoniae: Challenges for treatment, prevention and infection control. Expert Rev Anti Infect Ther. 2018;16(10):749–61. 10.1080/14787210.2018.1522249 30207815

[8] Lee GC, Burgess DS. Treatment of Klebsiella pneumoniae carbapenemase (KPC) infections: A review of published case series and case reports. Ann Clin Microbiol Antimicrob. 2012;11:32. 10.1186/1476-0711-11-32 PMC3552987 23234297

[9] Jacobs DM, Safir MC, Huang D, Minhaj F, Parker A, Rao GG. Triple combination antibiotic therapy for carbapenemase-producing Klebsiella pneumoniae: a systematic review. Ann Clin Microbiol Antimicrob. 2017;16(1):76. 10.1186/s12941-017-0249-2 PMC5702089 29178957

[10] Ferreira RL, da Silva BCM, Rezende GS, Nakamura-Silva R, Pitondo-Silva A, Campanini EB, et al. High Prevalence of Multidrug-Resistant Klebsiella pneumoniae Harboring Several Virulence and β-Lactamase Encoding Genes in a Brazilian Intensive Care Unit. Front Microbiol. 2019;22(9):3198. 10.3389/fmicb.2018.03198 PMC6349766 30723463

[11] Sikarwar AS, Batra HV. Prevalence of antimicrobial drug resistance of klebsiella pneumoniae in India. Int J Biosci Biochem Bioinf. 2011;1(3):211–5.

[12] Mulani MS, Kamble EE, Kumkar SN, Tawre MS, Pardesi KR. Emerging strategies to combat ESKAPE pathogens in the era of antimicrobial resistance: A review. Front Microbiol. 2019;10:539. 10.3389/fmicb.2019.00539 PMC6452778 30988669

[13] Santajit S, Indrawattana N. Mechanisms of antimicrobial resistance in ESKAPE pathogens. Biomed Res Int. 2016;2016:2475067. 10.1155/2016/2475067 PMC4871955 27274985

[14] Liu Y, Wan LG, Deng Q, Cao XW, Yu Y, Xu QF. First description of NDM-1-, KPC-2-, VIM-2- and IMP-4-producing Klebsiella pneumoniae strains in a single Chinese teaching hospital. Epidemiol Infect. 2015;143(2):376–84. 10.1017/S0950268814000995 PMC9206769 24762211

[15] Theuretzbacher U, Carrara E, Conti M, Tacconelli E. Role of new antibiotics for KPC-producing Klebsiella pneumoniae. J Antimicrob Chemother. 2021;76(Suppl 1):i47–54. 10.1093/jac/dkaa497 33534882

[16] Tooke CL, Hinchliffe P, Krajnc A, Mulholland AJ, Brem J, Schofield CJ, et al. Cyclic boronates as versatile scaffolds for KPC-2 beta-lactamase inhibition. RSC Med Chem. 2020;11(4):491–6. 10.1039/c9md00557a PMC7536818 33479650

[17] Yong D, Toleman MA, Giske CG, Cho HS, Sundman K, Lee K, et al. Characterization of a new metallo-beta-lactamase gene, bla(NDM-1), and a novel erythromycin esterase gene carried on a unique genetic structure in Klebsiella pneumoniae sequence type 14 from India. Antimicrob Agents Chemother. 2009;53(12):5046–54. 10.1128/AAC.00774-09 PMC2786356 19770275

[18] Hall BG, Barlow M. Revised Ambler classification of {beta}-lactamases. J Antimicrob Chemother. 2005;55(6):1050–1. 10.1093/jac/dki130 15872044

[19] Azargun R, Soroush Barhaghi MH, Samadi Kafil H, Ahangar Oskouee M, Sadeghi V, Memar MY, et al. Frequency of DNA gyrase and topoisomerase IV mutations and plasmid-mediated quinolone resistance genes among Escherichia coli and Klebsiella pneumoniae isolated from urinary tract infections in Azerbaijan, Iran. J Glob Antimicrob Resist. 2019;17:39–43. 10.1016/j.jgar.2018.11.003 30445211

[20] Haeili M, Javani A, Moradi J, Jafari Z, Feizabadi MM, Babaei E. MgrB Alterations Mediate Colistin Resistance in Klebsiella pneumoniae Isolates from Iran. Front Microbiol. 2017;8:2470. 10.3389/fmicb.2017.02470 PMC5741654 29326662

[21] Liu P, Chen S, Wu ZY, Qi M, Li XY, Liu CX. Mechanisms of fosfomycin resistance in clinical isolates of carbapenem-resistant Klebsiella pneumoniae. J Glob Antimicrob Resist. 2020;22:238–43. 10.1016/j.jgar.2019.12.019 32061879

[22] Li B, Zhao Y, Liu C, Chen Z, Zhou D. Molecular pathogenesis of Klebsiella pneumoniae. Future Microbiol. 2014;9(9):1071–81. 10.2217/fmb.14.48 25340836

[23] Liu EY, Chen JH, Lin JC, Wang CH, Fung CP, Ding YJ, et al. Cross-protection induced by highly conserved outer membrane proteins (Omps) in mice immunized with OmpC of Salmonella Typhi or OmpK36 of Klebsiella pneumoniae. Vaccine. 2022;40(18):2604–11. 10.1016/j.vaccine.2022.03.016 35331568

[24] Ye C, Li W, Yang Y, Liu Q, Li S, Zheng P, et al. Inappropriate use of antibiotics exacerbates inflammation through OMV-induced pyroptosis in MDR Klebsiella pneumoniae infection. Cell Rep. 2021;36(12):109750. 10.1016/j.celrep.2021.109750 34551309

[25] Wu LT, Guo MK, Ke SC, Lin YP, Pang YC, Nguyen HV, et al. Characterization of the genetic background of KPC-2-Producing Klebsiella pneumoniae with Insertion Elements Disrupting the ompK36 Porin Gene. Microb Drug Resist. 2020;26(9):1050–7. 10.1089/mdr.2019.0410 32283046

[26] Pulzova L, Navratilova L, Comor L. Alterations in outer membrane permeability favor drug-resistant phenotype of Klebsiella pneumoniae. Microb Drug Resist. 2017;23(4):413–20. 10.1089/mdr.2016.0017 27526080

[27] Nielsen LE, Snesrud EC, Onmus-Leone F, Kwak YI, Aviles R, Steele ED, et al. IS5 element integration, a novel mechanism for rapid in vivo emergence of tigecycline nonsusceptibility in Klebsiella pneumoniae. Antimicrob Agents Chemother. 2014;58(10):6151–6. 10.1128/AAC.03053-14 PMC4187979 25092708

[28] Tang M, Wei X, Wan X, Ding Z, Ding Y, Liu J. The role and relationship with efflux pump of biofilm formation in Klebsiella pneumoniae. Microb Pathog. 2020;147:104244. 10.1016/j.micpath.2020.104244 32437832

[29] Bialek-Davenet S, Lavigne JP, Guyot K, Mayer N, Tournebize R, Brisse S, et al. Differential contribution of AcrAB and OqxAB efflux pumps to multidrug resistance and virulence in Klebsiella pneumoniae. J Antimicrob Chemother. 2015;70(1):81–8. 10.1093/jac/dku340 25193085

[30] Bharatham N, Bhowmik P, Aoki M, Okada U, Sharma S, Yamashita E, et al. Structure and function relationship of OqxB efflux pump from Klebsiella pneumoniae. Nat Commun. 2021;12(1):5400. 10.1038/s41467-021-25679-0 PMC8437966 34518546

[31] Desai S, Sanghrajka K, Gajjar D. High adhesion and increased cell death contribute to strong biofilm formation in Klebsiella pneumoniae. Pathogens. 2019;8(4):277. 10.3390/pathogens8040277 PMC6963951 31805671

[32] Chung PY. The emerging problems of Klebsiella pneumoniae infections: carbapenem resistance and biofilm formation. FEMS Microbiol Lett. 2016;363(20):fnw219. 10.1093/femsle/fnw219 27664057

[33] Cepas V, López Y, Muoz E, Rolo D, Ardanuy C, Martí S, et al. Relationship between biofilm formation and antimicrobial resistance in gram-negative bacteria. Microb Drug Resist. 2019;25(1):72–9. Mary Ann Liebert, Inc. 10.1089/mdr.2018.0027 30142035

[34] Sirot D, Sirot J, Labia R, Morand A, Courvalin P, Darfeuille-Michaud A, et al. Transferable resistance to third-generation cephalosporins in clinical isolates of Klebsiella pneumoniae: identification of CTX-1, a novel beta-lactamase. J Antimicrob Chemother. 1987;20(3):323–34. 10.1093/jac/20.3.323 3316146

[35] Kliebe C, Nies BA, Meyer JF, Tolxdorff-Neutzling RM, Wiedemann B. Evolution of plasmid-coded resistance to broad-spectrum cephalosporins. Antimicrob Agents Chemother. 1985;28(2):302–7. 10.1128/aac.28.2.302 PMC180236 3879659

[36] Li CF, Tang HL, Chiou CS, Tung KC, Lu MC, Lai YC. Draft genome sequence of CTX-M-type beta-lactamase-producing Klebsiella quasipneumoniae subsp. similipneumoniae isolated from a Box turtle. J Glob Antimicrob Resist. 2018;12:235–6. 10.1016/j.jgar.2017.12.012 29291945

[37] Bradford PA. Extended-spectrum beta-lactamases in the 21st century: characterization, epidemiology, and detection of this important resistance threat. Clin Microbiol Rev. 2001;14(4):933. 10.1128/CMR.14.4.933-951.2001 PMC89009 11585791

[38] Evans BA, Amyes SGB. OXA β-lactamases. Clin Microbiol Rev. 2014;27(2):241–63. 10.1128/CMR.00117-13 PMC3993105 24696435

[39] Li P, Shen K, Zhang Y, Ying J, Zhu T, Liu Y, et al. Characterization of a Novel blaKLUC Variant With Reduced β-Lactam Resistance From an IncA/C Group Plasmid in a Clinical Klebsiella pneumoniae Isolate. Front Microbiol. 2018;15(9):1908. 10.3389/fmicb.2018.01908 PMC6104158 30158920

[40] Slama P, Deny P, Labia R, Philippon A. A structure-based classification of class a beta-lactamases, a broadly diverse family of enzymes. Clin Microbiol Rev. 2016;29(1):29–57. 10.1128/CMR.00019-15 PMC4771212 26511485

[41] Lee CR, Lee JH, Park KS, Kim YB, Jeong BC, Lee SH. Global dissemination of carbapenemase-producing klebsiella pneumoniae: Epidemiology, genetic context, treatment options, and detection methods. Front Microbiol. 2016;7:895. 10.3389/fmicb.2016.00895 PMC4904035 27379038

[42] Papp-Wallace KM, Bethel CR, Distler AM, Kasuboski C, Taracila M, Bonomo RA. Inhibitor resistance in the KPC-2 beta-lactamase, a preeminent property of this class A beta-lactamase. Antimicrob Agents Chemother. 2010;54(2):890–7. 10.1128/AAC.00693-09 PMC2812178 20008772

[43] Bush K. Bench-to-bedside review: The role of beta-lactamases in antibiotic-resistant Gram-negative infections. Crit Care. 2010;14(3):224. 10.1186/cc8892 PMC2911681 20594363

[44] Jacoby GA. AmpC beta-lactamases. Clin Microbiol Rev. 2009;22(1):161–82. 10.1128/CMR.00036-08 PMC2620637 19136439

[45] Opal SM, Medeiros AA. Molecular mechanisms of antibiotic resistance in bacteria. Mandell, Douglas, and Bennett’s Principles and Practice of Infectious Diseases. 8th edn. Vol. 1. Issue 2; 2015. p. 235–51.

[46] Doi Y, Wachino JI, Arakawa Y. Aminoglycoside resistance: The emergence of acquired 16S ribosomal RNA methyltransferases. Infect Dis Clin North Am. 2016;30(2):523–37. 10.1016/j.idc.2016.02.011 PMC4878400 27208771

[47] Poulikakos P, Falagas ME. Aminoglycoside therapy in infectious diseases. Expert Opin Pharmacother. 2013;14(12):1585–97. 10.1517/14656566.2013.806486 23746121

[48] Galimand M, Courvalin P, Lambert T. Plasmid-mediated high-level resistance to aminoglycosides in enterobacteriaceae due to 16S rRNA methylation. Antimicrob Agents Chemother. 2003;47(8):2565–71. 10.1128/AAC.47.8.2565-2571.2003 PMC166065 12878520

[49] Srinivasan VB, Venkataramaiah M, Mondal A, Vaidyanathan V, Govil T, Rajamohan G. Functional characterization of a novel outer membrane porin KpnO, regulated by PhoBR two-component system in Klebsiella pneumoniae NTUH-K2044. PLoS One. 2012;7(7):e41505. 10.1371/journal.pone.0041505 PMC3405095 22848515

[50] Nam YS, Cho SY, Yang HY, Park KS, Jang JH, Kim YT, et al. Investigation of mutation distribution in DNA gyrase and topoisomerase IV genes in ciprofloxacin-non-susceptible Enterobacteriaceae isolated from blood cultures in a tertiary care university hospital in South Korea, 2005–2010. Int J Antimicrob Agents. 2013;41(2):126–9. 10.1016/j.ijantimicag.2012.10.004 23265914

[51] Martinez-Martinez L, Hernández-Allés S, Albertí S, Tomás JM, Benedi VJ, Jacoby GA. In vivo selection of porin-deficient mutants of Klebsiella pneumoniae with increased resistance to cefoxitin and expanded-spectrum-cephalosporins. Antimicrob Agents Chemother. 1996;40(2):342. 10.1128/aac.40.2.342 PMC163113 8834877

[52] Mazzariol A, Zuliani J, Cornaglia G, Rossolini GM, Fontana R. AcrAB efflux system: Expression and contribution to fluoroquinolone resistance in Klebsiella spp. Antimicrob Agents Chemother. 2002;46(12):3984–6. 10.1128/AAC.46.12.3984-3986.2002 PMC132751 12435706

[53] Ping Y, Ogawa W, Kuroda T, Tsuchiya T. Gene cloning and characterization of KdeA, a multidrug efflux pump from Klebsiella pneumoniae. Biol Pharm Bull. 2007;30(10):1962–4196. 10.1248/bpb.30.1962 17917272

[54] Ruiz E, Saenz Y, Zarazaga M, Rocha-Gracia R, Martinez-Martinez L, Arlet G, et al. qnr, aac(6’)-Ib-cr and qepA genes in Escherichia coli and Klebsiella spp.: genetic environments and plasmid and chromosomal location. J Antimicrob Chemother. 2012;67(4):886–97. 10.1093/jac/dkr548 22223228

[55] da Silva DM, Faria-Junior C, Nery DR, de Oliveira PM, Silva LD, Alves EG, et al. Insertion Sequences Disrupting mgrB in Carbapenem-Resistant Klebsiella pneumoniae Strains in Brazil. J Glob Antimicrob Resist. 2020;24:53–7. 10.1016/j.jgar.2020.11.003 33246210

[56] Laurent P, Aurélie J, Séverine B, Maria-Virginia V, Melda O, Salih T, et al. The mgrB gene as a key target for acquired resistance to colistin in Klebsiella pneumoniae. J Antimicrob Chemother. 2015;1:75–80. 10.1093/jac/dku323 25190723

[57] Cannatelli A, Santos-Lopez A, Giani T, Gonzalez-Zorn B, Rossolini GM. Polymyxin Resistance Caused by mgrB Inactivation Is Not Associated with Significant Biological Cost in Klebsiella pneumoniae. Antimicrob Agents Chemother. 2015;59(5):2898–900. 10.1128/AAC.04998-14 PMC4394794 25691629

[58] Lv J, Li R, Xie M, Chen S, Chan E. Complete genetic analysis of plasmids carrying mcr-1 and other resistance genes in an Escherichia coli isolate of animal origin. J Antimicrob Chemother. 2017;72(3):696–9. 10.1093/jac/dkw509 27999050

[59] Lai CC, Chuang YC, Chen CC, Tang HJ. Coexistence of MCR-1 and NDM-9 in a clinical carbapenem-resistant Escherichia coli isolate. Int J Antimicrob Agents. 2017;49(4):517–23. 10.1016/j.ijantimicag.2017.02.001 28219753

[60] Mcgann P, Snesrud E, Maybank R, Corey B, Schaecher KE. Escherichia coli harboring mcr-1 and blaCTX-M on a novel IncF plasmid: First report of mcr-1 in the United States. Antimicrob Agents Chemother. 2016;60(7):4420–4. 10.1128/AAC.01103-16 PMC4914657 27230792

[61] Sekyere JO, Govinden U, Bester LA, Essack SY. Colistin and tigecycline resistance in carbapenemase‐producing Gram‐negative bacteria: emerging resistance mechanisms and detection methods. J Appl Microbiol. 2016;121(3):601–17. 10.1111/jam.13169 27153928

[62] Villa L, Feudi C, Fortini D, Garcia-Fernandez A, Carattoli A. Genomics of KPC-producing Klebsiella pneumoniae sequence type 512 clone highlights the role of RamR and Ribosomal S10 protein mutations in conferring tigecycline resistance. Antimicrob Agents Chemother. 2014;58(3):1707–12. 10.1128/AAC.01803-13 PMC3957836 24379204

[63] Liu P, Chen S, Wu ZY, Qi M, Liu CX. Mechanisms of fosfomycin resistance in clinical isolates of carbapenem-resistant Klebsiella pneumoniae. J Glob Antimicrob Resist. 2020;22:238–43. 10.1016/j.jgar.2019.12.019 32061879

[64] Sirot D, Sirot J, Labia R, Morand A, Courvalin P. Transferable resistance to third-generation cephalosporins in clinical isolates of Klebsiella pneumoniae: identification of CTX-1, a novel β-lactamase. Jantimicrobchemother. 1987;20(3):323–34. 10.1093/jac/20.3.323 3316146

[65] Bush K, Jacoby GA, Medeiros AA. A functional classification scheme for beta-lactamases and its correlation with molecular structure. Antimicrob Agents Chemother. 1995;39(6):1211–33. 10.1128/aac.39.6.1211 PMC162717 7574506

[66] Breurec S, Guessennd N, Timinouni M, Le TA, Cao V, Ngandjio A, et al. Klebsiella pneumoniae resistant to third-generation cephalosporins in five African and two Vietnamese major towns: Multiclonal population structure with two major international clonal groups, CG15 and CG258. Clin Microbiol Infect. 2013;19(4):349–55. 10.1111/j.1469-0691.2012.03805.x 22390772

[67] Ørjan S, Umaer N, Ståle T, Harald SD, Annette O, Reidar H, et al. Emergence of clonally related Klebsiella pneumoniae isolates of sequence type 258 producing plasmid-mediated KPC carbapenemase in Norway and Sweden. J Antimicrob Chemother. 2009;4:654–8. 10.1093/jac/dkp018 19218573

[68] Andrade LN, Aldc D, Curiao T, Baquero F, Cantón R, Coque TM. Clonal Complex 258, the most frequently found multilocus sequence type complex in KPC-2-producing Klebsiella pneumoniae isolated in Brazilian Hospitals. Antimicrob Agents Chemother. 2012;56(8):4563–4. author reply 4565. 10.1128/AAC.00219-12 PMC3421559 22826287

[69] Baraniak A, Izdebski R, Herda M, Fiett J, Hryniewicz W, Gniadkowski M, et al. Emergence of Klebsiella pneumoniae ST258 with KPC-2 in Poland. Antimicrob Agents Chemother. 2009;53(10):4565–7. 10.1128/AAC.00436-09 PMC2764197 19620323

[70] Garcia-Fernandez A, Villa L, Carta C, Venditti C, Giordano A, Venditti M, et al. Klebsiella pneumoniae ST258 producing KPC-3 identified in italy carries novel plasmids and OmpK36/OmpK35 porin variants. Antimicrob Agents Chemother. 2012;56(4):2143–5. 10.1128/AAC.05308-11 PMC3318348 22252815

[71] Kitchel B, Rasheed JK, Patel JB, Srinivasan A, Navon-Venezia S, Carmeli Y, et al. Molecular epidemiology of KPC-producing Klebsiella pneumoniae isolates in the United States: clonal expansion of multilocus sequence type 258. Antimicrob Agents Chemother. 2009;53(8):3365–70. 10.1128/AAC.00126-09 PMC2715580 19506063

[72] Liu P, Li P, Jiang X, Bi D, Xie Y, Tai C, et al. Complete genome sequence of Klebsiella pneumoniae subsp. pneumoniae HS11286, a multidrug-resistant strain isolated from human sputum. J Bacteriol. 2012;194(7):1841–2. 10.1128/JB.00043-12 PMC3302456 22408243

[73] Naas T, Cuzon G, Truong HV, Nordmann P. Role of ISKpn7 and deletions in blaKPC gene expression. Antimicrob Agents Chemother. 2012;56(9):4753–9. 10.1128/AAC.00334-12 PMC3421896 22733068

[74] Chmelnitsky I, Shklyar M, Leavitt A, Sadovsky E, Navon-Venezia S, Ben Dalak M, et al. Mix and match of KPC-2 encoding plasmids in Enterobacteriaceae-comparative genomics. Diagn Microbiol Infect Dis. 2014;79(2):255–60. 10.1016/j.diagmicrobio.2014.03.008 24743043

[75] Ripabelli G, Sammarco ML, Salzo A, Scutella M, Felice V, Tamburro M. New Delhi metallo-beta-lactamase (NDM-1)-producing Klebsiella pneumoniae of sequence type ST11: first identification in a hospital of central Italy. Lett Appl Microbiol. 2020;71(6):652–9. 10.1111/lam.13384 32916001

[76] Papp-Wallace KM, Bethel CR, Distler AM, Kasuboski C, Taracila M, Bonomo RA. Inhibitor Resistance in the KPC-2 β-Lactamase, a Preeminent Property of This Class A β-Lactamase. Antimicrob Agents Chemother. 2010;54(2):890–7. 10.1128/AAC.00693-09 PMC2812178 20008772

[77] Cristina ML, Alicino C, Sartini M, Faccio V, Spagnolo AM, Bono VDL, et al. Epidemiology, management, and outcome of carbapenem-resistant Klebsiella pneumoniae bloodstream infections in hospitals within the same endemic metropolitan area. J Infect Public Health. 2017;11(2):171–7. 10.1016/j.jiph.2017.06.003 28668656

[78] Krause KM, Serio AW, Kane TR, Connolly LE. Aminoglycosides: An overview. Cold Spring Harb Perspect Med. 2016;6(6):a027029. 10.1101/cshperspect.a027029 PMC4888811 27252397

[79] Redgrave LS, Sutton SB, Webber MA, Piddock LJ. Fluoroquinolone resistance: mechanisms, impact on bacteria, and role in evolutionary success. Trends Microbiol. 2014;22(8):438–45. 10.1016/j.tim.2014.04.007 24842194

[80] Wong M, Chan E, Chen S. Evolution and Dissemination of OqxAB-like efflux pumps, an emerging quinolone resistance determinant among members of enterobacteriaceae. Antimicrob Agents Chemother. 2015;59(6):3290–7. 10.1128/AAC.00310-15 PMC4432112 25801572

[81] Zheng JX, Lin ZW, Sun X, Lin WH, Chen Z, Wu Y, et al. Overexpression of OqxAB and MacAB efflux pumps contributes to eravacycline resistance and heteroresistance in clinical isolates of Klebsiella pneumoniae. Emerg Microbes Infect. 2018;7(1):139–46. 10.1038/s41426-018-0141-y PMC6070572 30068997

[82] Ruiz E, Sáenz Y, Zarazaga M, Rocha-Gracia R, Martínez-Martínez L, Arlet G, et al. qnr, aac(6′)-Ib-cr and qepA genes in Escherichia coli and Klebsiella spp.: genetic environments and plasmid and chromosomal location. J Antimicrob Chemother. 2012;67(4):886–97. 10.1093/jac/dkr548 22223228

[83] Fabrega A, Madurga S, Giralt E, Vila J. Mechanism of action of and resistance to quinolones. Microb Biotechnol. 2009;2(1):40–61. 10.1111/j.1751-7915.2008.00063.x PMC3815421 21261881

[84] Anastasia A, Flora K, Garifalia P, Evangelos K, Irene G, Evangelos P, et al. Colistin-resistant isolates of Klebsiella pneumoniae emerging in intensive care unit patients: first report of a multiclonal cluster. J Antimicrob Chemother. 2007;59(4):786–92. 10.1093/jac/dkl562 17307769

[85] Liu X, Liu H, Li Y, Hao C. High prevalence of β-lactamase and plasmid-mediated quinolone resistance genes in extended-spectrum cephalosporin-resistant Escherichia coli from Dogs in Shaanxi, China. Front Microbiol. 2016;7:1843–51. 10.3389/fmicb.2016.01843 PMC5111280 27899921

[86] Guillard T, Jong AD, Limelette A, Lebreil AL, Champs CD. Characterization of quinolone resistance mechanisms in Enterobacteriaceae recovered from diseased companion animals in Europe. Vet Microbiol. 2016;194:23–9. 10.1016/j.vetmic.2015.11.033 26701806

[87] Beabout K, Hammerstrom TG, Perez AM, Magalh EB, Prater AG, Clements TP, et al. The ribosomal S10 protein is a general target for decreased tigecycline susceptibility. Antimicrob Agents Chemother. 2015;59(9):5561–6. 10.1128/AAC.00547-15 PMC4538488 26124155

[88] Lupien A, Gingras H, Leprohon P, Ouellette M. Induced tigecycline resistance inStreptococcus pneumoniae mutants reveals mutations in ribosomal proteins and rRNA. J Antimicrob Chemother. 2015;70(11):2973–80. 10.1093/jac/dkv211 26183184

[89] Raz R. Fosfomycin: An old—new antibiotic. Clin Microbiol Infect. 2012;18(1):4–7. 10.1111/j.1469-0691.2011.03636.x 21914036

[90] Kurabayashi K, Tanimoto K, Fueki S, Tomita H, Hirakawa H. Elevated expression of GlpT and UhpT via FNR activation contributes to increased fosfomycin susceptibility in escherichia coli under anaerobic conditions. Antimicrob Agents Chemother. 2015;59(10):6352–60. 10.1128/AAC.01176-15 PMC4576066 26248376

[91] Liu Y, Cheng Y, Yang H, Hu L, Cheng J, Ye Y, et al. Characterization of extended-spectrum β-lactamase genes of shigella flexneri isolates with Fosfomycin resistance from patients in China. Ann Lab Med. 2017;37(5):415–9. 10.3343/alm.2017.37.5.415 PMC5500740 28643490

[92] Cao XL, Shen H, Xu YY, Xu XJ, Zhang ZF, Cheng L, et al. High prevalence of fosfomycin resistance gene fosA3 in bla CTX-M-harbouring Escherichia coli from urine in a Chinese tertiary hospital during 2010-2014. Epidemiol Infect. 2017;145(4):1–7. 10.1017/S0950268816002879 PMC9507793 27938421

[93] Falagas ME, Athanasaki F, Voulgaris GL, Triarides NA, Vardakas KZ. Resistance to fosfomycin: Mechanisms, frequency and clinical consequences. Int J Antimicrob Agents. 2018;53(1):22–8. 10.1016/j.ijantimicag.2018.09.013 30268576

[94] Levin-Reisman I, Ronin I, Gefen O, Braniss I, Shoresh N, Balaban NQ. Antibiotic tolerance facilitates the evolution of resistance. Science. 2017;355(6327):826–30. 10.1126/science.aaj2191 28183996

[95] Kim JS, Wood TK, Blaser MJ. Tolerant, growing cells from nutrient shifts are not persister cells. Mbio. 2017;8(2):e00354-17. 10.1128/mBio.00354-17 PMC5395667 28420737

[96] Pontes MH, Groisman EA. Slow growth determines nonheritable antibiotic resistance in Salmonella enterica. Sci Signal. 2019;12(592):eaax3938-45. 10.1126/scisignal.aax3938 PMC7206539 31363068

[97] Ackermann M. A functional perspective on phenotypic heterogeneity in microorganisms. Nat Rev Microbiol. 2015;13(8):497–508. 10.1038/nrmicro3491 26145732

[98] Balaban NQ, Merrin J, Chait R, Kowalik L, Leibler S. Bacterial persistence as a phenotypic switch. Science. 2004;305(5690):1622–5. 10.1126/science.1099390 15308767

[99] Dhar N, McKinney JD. Microbial phenotypic heterogeneity and antibiotic tolerance. Curr Opin Microbiol. 2007;10(1):30–8. 10.1016/j.mib.2006.12.007 17215163

[100] Ren H, He X, Zou X, Wang G, Li S, Wu Y. Gradual increase in antibiotic concentration affects persistence of Klebsiella pneumoniae. J Antimicrob Chemother. 2015;70(12):3267–72. 10.1093/jac/dkv251 26311842

[101] Salou M, Ekoue-Toulan DE, Dossim S, Agbonon A. In vitro activities of aqueous and hydro-ethanolic extracts of ocimum gratissimum on escherichia coli esbl, klebsiella pneumoniae esbl and methicillin- resistant staphylococcus aureus. Acad J. 2019;13(3):55–9.

[102] Principi N, Silvestri E, Esposito S. Advantages and limitations of bacteriophages for the treatment of bacterial infections. Front Pharmacol. 2019;10:513–20. 10.3389/fphar.2019.00513 PMC6517696 31139086

[103] Lee NY, Ko WC, Hsueh PR. Nanoparticles in the treatment of infections caused by multidrug-resistant organisms. Front Pharmacol. 2019;10:1153–3. 10.3389/fphar.2019.01153 PMC6787836 31636564

[104] Liu C, Zhou Y, Wang L, Han L, Lei J, Ishaq HM, et al. Photodynamic inactivation of Klebsiella pneumoniae biofilms and planktonic cells by 5-aminolevulinic acid and 5-aminolevulinic acid methyl ester. Lasers Med. 2016;31(3):557–65. 10.1007/s10103-016-1891-1 26886586

[105] Dias LP, Souza P, Oliveira J, Vasconcelos IM, Araújo N. RcAlb-PepII, a synthetic small peptide bioinspired in the 2S albumin from the seed cake of Ricinus communis, is a potent antimicrobial agent against Klebsiella pneumoniae and Candida parapsilosis. Biochim Biophys Acta-Biomembr 2020;1862(2):183092–8. 10.1016/j.bbamem.2019.183092 31678367

